# Identification of a novel mutation in the *CLCN7* gene in pediatric osteopetrosis: case report

**DOI:** 10.3389/fped.2025.1549961

**Published:** 2025-04-10

**Authors:** Aoshuang Jiang, Tianping Chen, Nan Wei, Chenglin Zhu, Jie Wang, Hongjun Liu, Min Wang

**Affiliations:** Department of Hematology and Oncology, Anhui Provincial Children's Hospital (Anhui Hospital, Pediatric Hospital of Fudan University), Hefei Anhui, China

**Keywords:** osteopetrosis, *CLCN7* gene, variant, bioinformatics analysis, children

## Abstract

Osteopetrosis, also known as osteosclerosis and marble-bone disease, is a rare genetic metabolic bone disorder caused by the dysplasia or dysfunction of osteoclasts, usually caused by variants of chloride voltage-gated channel 7 (*CLCN7)* gene. We retrospectively analyzed the clinical data of two children with osteopetrosis and their families. Whole-exome sequencing (WES) was used for genetic analysis, and Sanger sequencing confirmed possible pathogenic variants. In family 1, the proband harbored a novel mutation c.2351G>C (p.R784T) in *CLCN7* gene. The initial symptom of proband 1 was a post-traumatic fracture, and imaging features was “sandwich cake” -like changes. In family 2, the proband harbored previously reported compound heterozygous variants in *CLCN7* gene: c.899C>T (p.A300V) and c.1534G>A (p.G512R). Among them, c.1534G>A (p.G512R) was only recorded in clinvar and no reports of protein function prediction. The initial symptom of proband 2 was cough, and imaging features was “sandwich vertebrae”. Our study expands the mutation spectrum of the *CLCN7* gene and provides new insights into the pathogenesis of osteopetrosis.

## 1 Introduction

Osteopetrosis, also known as osteosclerosis or marble bone disease, is a rare genetic metabolic bone disease characterized by defective osteoclast function, leading to abnormal bone metabolism and a systemic increase in bone density (1–3). The expansion of bone into the bone marrow cavity and cranial nerve foramina can impair blood and nerve function, respectively. The former may result in severe anemia, extramedullary hematopoiesis leading to bleeding, frequent infections, and hepatosplenomegaly, while the latter can cause blindness, deafness, and nerve paralysis (4). Osteopetrosis is classified based on its inheritance patterns into autosomal recessive osteopetrosis (ARO), autosomal dominant osteopetrosis (ADO), and X-linked osteopetrosis (XLO).

*CLCN7* gene variants can cause either recessive or dominant disease. Biallelic variants result in severe osteopetrosis, while the most common defect in *CLCN7* is a heterozygous missense variant. Approximately 75% of ADO patients, 17% of ARO patients, and all known patients of intermediate autosomal osteopetrosis (IAO) are linked to *CLCN7* variants (5–8). *CLCN7* gene variants are related to a broad spectrum of phenotypes, ranging from no clinical symptoms to bone marrow hematopoietic failure, and even life-threatening conditions (9). Over 150 variants in *CLCN7* are known (http://www.hgmd.cf.ac.uk/), but their relationship with clinical symptoms and variability in initial presentation is unclear.

In this study, we conducted a comprehensive analysis of the clinical phenotypes, genotypes, bioinformatics analysis of two children with osteopetrosis and their families. We identified a novel mutation c.2351G>C (p.R784T) and two reported mutation c.899C>T (p.A300V) and c.1534G>A (p.G512R) in *CLCN7* gene. Protein domain prediction and structure analysis for the three variants of CLCN7 were performed. Our results not only expanded the mutation spectrum of the *CLCN7* gene but also provided preliminary insights into the molecular pathogenesis associated with these variants.

## 2 Case presentation

### 2.1 Clinical data and treatment outcome

**Figure 1:**
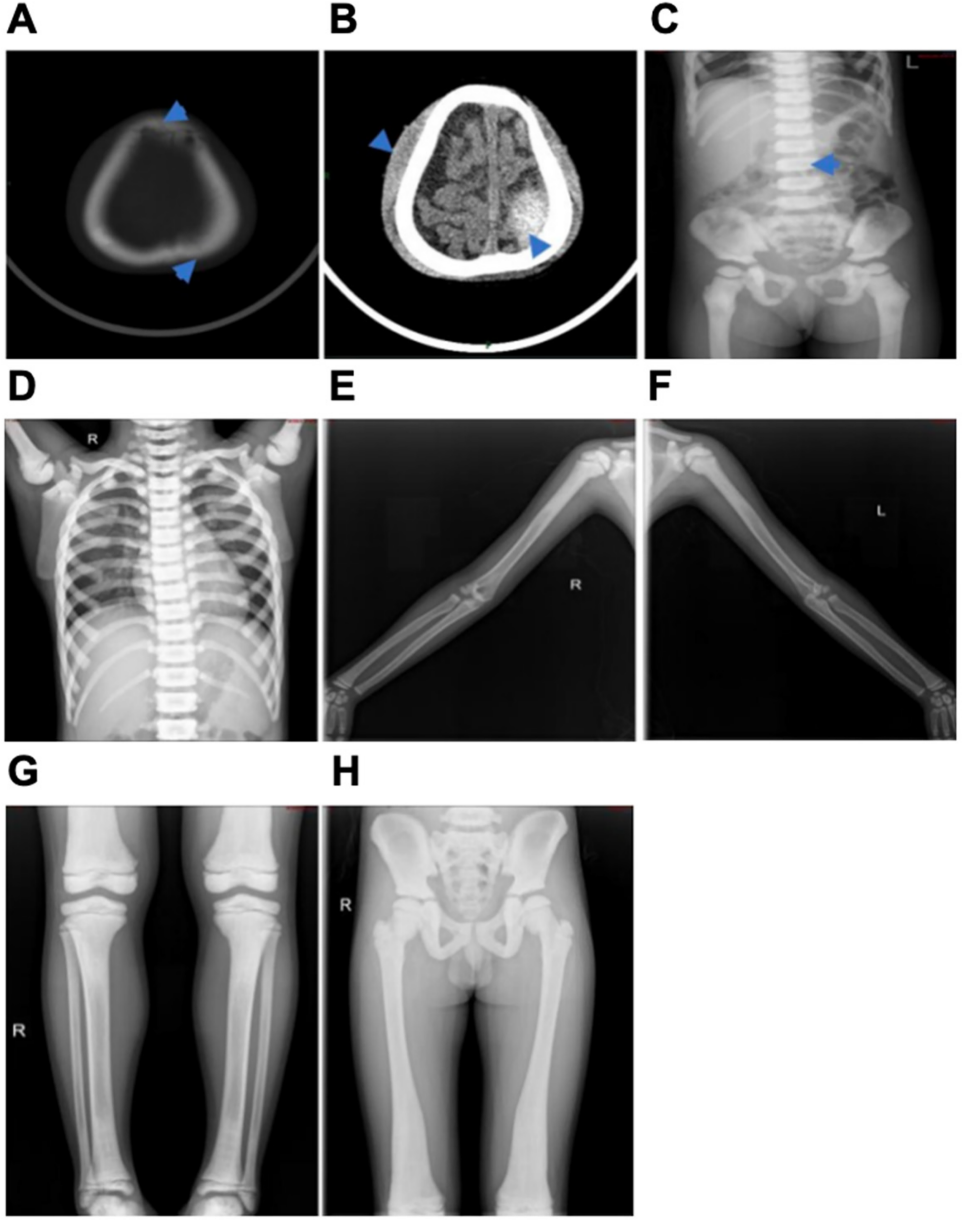
Abdominal x-ray film of the proband of patient 1 **(A–C)** and x-rays of the proband of patient 2 (D-H). **(A)** The top arrow represents a frontal fracture and the bottom arrow represents a left parietal fracture. **(B)** The top arrow represents a scalp hematoma and the bottom arrow represents epidural blood. **(C)** Arrows represent diffuse increased density of the spine, ribs, pelvis, and proximal femur, marked hardening of the upper and lower vertebrae, flattening of the vertebrae, and “sandwich-like” changes. **(D)** The diffuse density of the bones in the film increased, and the vertebral body was “sandwich-like” change. **(E,F)** The bone density of both humerus increased, the bone cortex thickened, and the medullary cavity narrowed. **(G)** The distal femur and proximal tibia showed “flakelike” changes. **(H)** The bone density of pelvic bones increased significantly, the bone cortex thickened, the bilateral femoral bone marrow cavity was not clear, and the iliac bone showed “bone in bone”.

Patient 1, a 3-year-old girl, visited the doctor due to nausea and vomiting for 2 days after head trauma. Her parents were not closely related and both had normal phenotypes. The first symptom of the patient was a head fracture following trauma, accompanied by a 6 × 7 cm mass at the top of the left forehead that was soft to the touch, well-defined, and demonstrated positive wave sensation. Laboratory findings showed white blood cells at 7.56 × 10^9^/L, hemoglobin at 89 g/L, platelets at 353 × 10^9^/L, blood calcium at 2.33 mmol/L, and blood phosphorus at 1.36 mmol/L. Other results included creatine kinase at 215 IU/L, creatine kinase isoenzyme at 102 U/L, lactate dehydrogenase at 548 IU/L, alkaline phosphatase at 92 IU/L (Table 1). Cranial CT scans showed left parietal and frontal bone fractures with epidural bleeding and scalp hematoma, and increased bone density in the skull and maxillofacial region. EEG topographic maps showed no obvious abnormalities. x-ray examinations revealed flattened vertebral bodies with “sandwich cake” -like changes, extensive increased bone density, and hardening, along with “halo-like” changes in the iliac bone wings (Figure 1A–C). The patient received conservative treatment and was discharged upon improvement, diagnosed as autosomal dominant osteopetrosis type II (ADOII). During follow-up, recurrent anemia, splenomegaly, a fibula fracture, and abnormal vision were noted. Allogeneic hematopoietic stem cell transplantation (HSCT) was performed in October 2023, and blood transfusions were required post-transplantation until the last follow-up.

**Table 1:** Clinical characteristics and laboratory tests of the probands.

Patient members	Sex	Age (years)	WBC (10^9^/L)	Hb (g/L)	PLT (10^9^/L)	Ca (mmol/L)	*P* (mmol/L)	CK (IU/L)	CKMB (U/L)	LDH (IU/L)	ALP (IU/L)	Bone fracture	Other clinical characteristics
F1	F	3	7.56	89	353	2.33	1.3	215	102	548	92	Yes	Anemia, bnormal vision
F2	M	8	6.18	119	335	2.22	1.69	368	123	572	212	No	No

WBC, white blood cell (reference range:×10^9^/L); Hb, hemoglobin (reference range:g/L); PLT, platelets (reference range:×10^9^/L); Ca, calcium (reference range:2.2–2.6 mmol/L); P, phosphorus (reference range: 0.8–1.6 mmol/L); CK, creatine kinase (reference range:0–210 IU/L); CKMB, creatine kinase isoenzyme (reference range:0–30 U/L; LDH, lactate dehydrogenase (reference range:80–285 IU/L); ALP, alkaline phosphate (reference range:30–500 IU/L).

Patient 2, an 8-year-old boy, initially presented with a week-long cough. His parents were not closely related, and their phenotype was normal. However, his 17-year-old sister had a history of optic atrophy and skeletal dysplasia. The first symptom of the proband was a cough, and chest radiograph revealed “sandwich vertebrae” -like change (Figure 1D). Laboratory results showed white blood cell count of 6.18 × 10^9^/L, hemoglobin of 119 g/L, platelet count of 335 × 10^9^/L, blood calcium of 2.22 mmol/L, blood phosphorus of 1.69 mmol/L. Other values included creatine kinase at 368 IU/L, creatine kinase isoenzyme at 123 U/L, lactate dehydrogenase at 572 IU/L, alkaline phosphatase at 212 IU/L (Table 1). X-ray examination of the long bones in the limbs showed irregular shapes of multiple bones, increased bone density, and typical “halo-like” changes in the iliac bone wings (Figures 1E–H). Cranial MRI indicated decreased T1W1 bone signal in the cranial diploid and slope, with no abnormal signal in the brain parenchyma. No abnormal clinical phenotype associated with *CLCN7*-related ARO was observed in the proband, and IAO was diagnosed. During the follow-up of patient 2, hemoglobin levels fluctuated between 50 and 102 g/L, and platelet counts ranged from 53 to 210 × 10^9^/L. The patient required blood transfusions approximately every 2–3 months, with the volume of red blood cells transfused being 0.5–1.0 units per 10 kg each time. Additionally, the patient exhibited progressive splenomegaly. Comprehensive peripheral blood examination results suggested that the patient's anemia was progressively worsening, necessitating frequent transfusion support. Moreover, the patient showed signs of extramedullary hematopoiesis, primarily manifested as progressive splenomegaly. These symptoms were all considered to be related to bone marrow hematopoietic failure.

### 2.2 Genetic analysis

Whole exome sequencing (WES) was performed on both patients based on their clinical phenotypes and initial diagnoses. Subsequent genetic investigation revealed the presence of missense variants in the *CLCN7* gene (NM_001287.6) in both patients. No additional variants were found in genes known to cause osteopetrosis.

**Figure 2:**
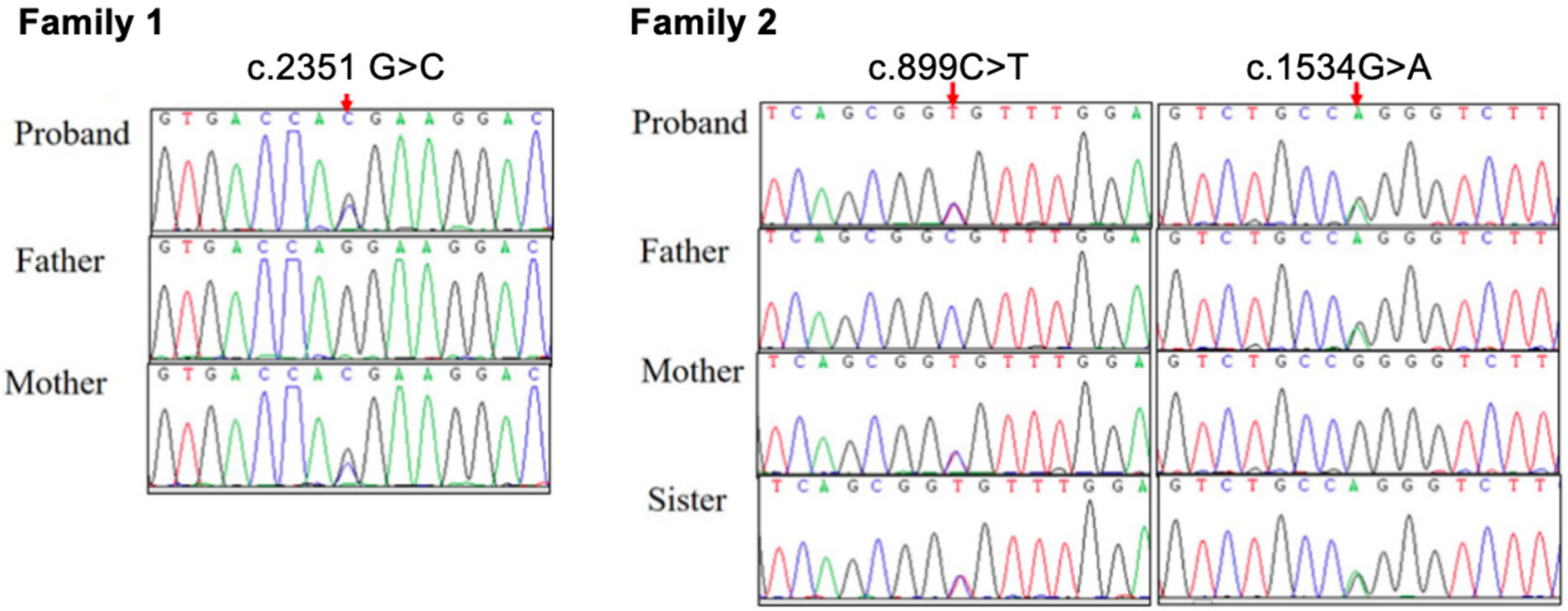
Sanger sequence of *CLCN7* gene variants in two families with osteopetrosis. The proband of family 1 was maternally inherited the variant of c.2351G>C (p.R784T). The proband of family 2 and his sister carry compound heterozygous variants: c.899C>T (p.A300V) inherited from their mother, and c.1534G>A (p.G512R) inherited from their father.

In family 1, a heterozygous missense variant c.2351G>C (p.R784T) in exon 25 was detected in patient 1 and her asymptomatic mother (Figure 2). According to American College of Medical Genetics and Genomics (ACMG) guidelines, the variant c.2351G>C (p.R784T) is rated as “likely pathogenic” (PM1 + PM2 + PM5+ PP3 + PP4). This variant is novel and has not been reported in Database of Single Nucleotide Polymorphisms (dbSNP), the 1,000 Genomes Project, Exome Aggregation Consortium (EXAC), Exome Sequencing Project (ESP), Genome Aggregation Database (gnomAD), Clinical Variation Database (ClinVar) or Human Gene Mutation Database (HGMD). Multiple bioinformatics tools (Provean: −5.37, SIFT: 0.0, PolyPhen2_HDIV: 1.0, PolyPhen2_HVAR: 1.0, Mutationtaster: 1, M-cap: 0.7986, REVEL: 0.946) predicted the c.2351G>C variant to be deleterious. A different variant at the same position, R784W, has been reported in the HGMD database as “Disease-causing Mutation (DM)” in four patients with ADOII.

In family 2, patient 2 had compound heterozygous missense variants: c.899C>T (p.A300 V) in exon 10 inherited from his mother, and c.1534G>A (p.G512R) in exon 17 inherited from his father. Sanger sequencing confirmed that both parents carried these variants in a heterozygous state, while patient 2 and his older sister were identified as compound heterozygous for these variants, consistent with autosomal recessive inheritance (Figure 2). The c.899C>T variant has been previously reported in two siblings with the IARO phenotype and multiple bioinformatics tools (Provean: −3.86, SIFT: 0.0, PolyPhen2_HDIV: 1.0, PolyPhen2_HVAR: 0.999, Mutationtaster: 1, M-cap: 0.6205, REVEL: 0.839, CADD: 26.1) predicted the variant to be deleterious. The c.1534G>A variant has been recorded in clinvar and multiple bioinformatics tools (Provean: −7.79, SIFT: 0.0, PolyPhen2_HDIV: 1.0, PolyPhen2_HVAR: 1.0, Mutationtaster: 1, M-cap: 0.8849, REVEL: 0.97, CADD: 26.7) predicted the variant to be deleterious.

### 2.3 Protein modelling

**Figure 3:**
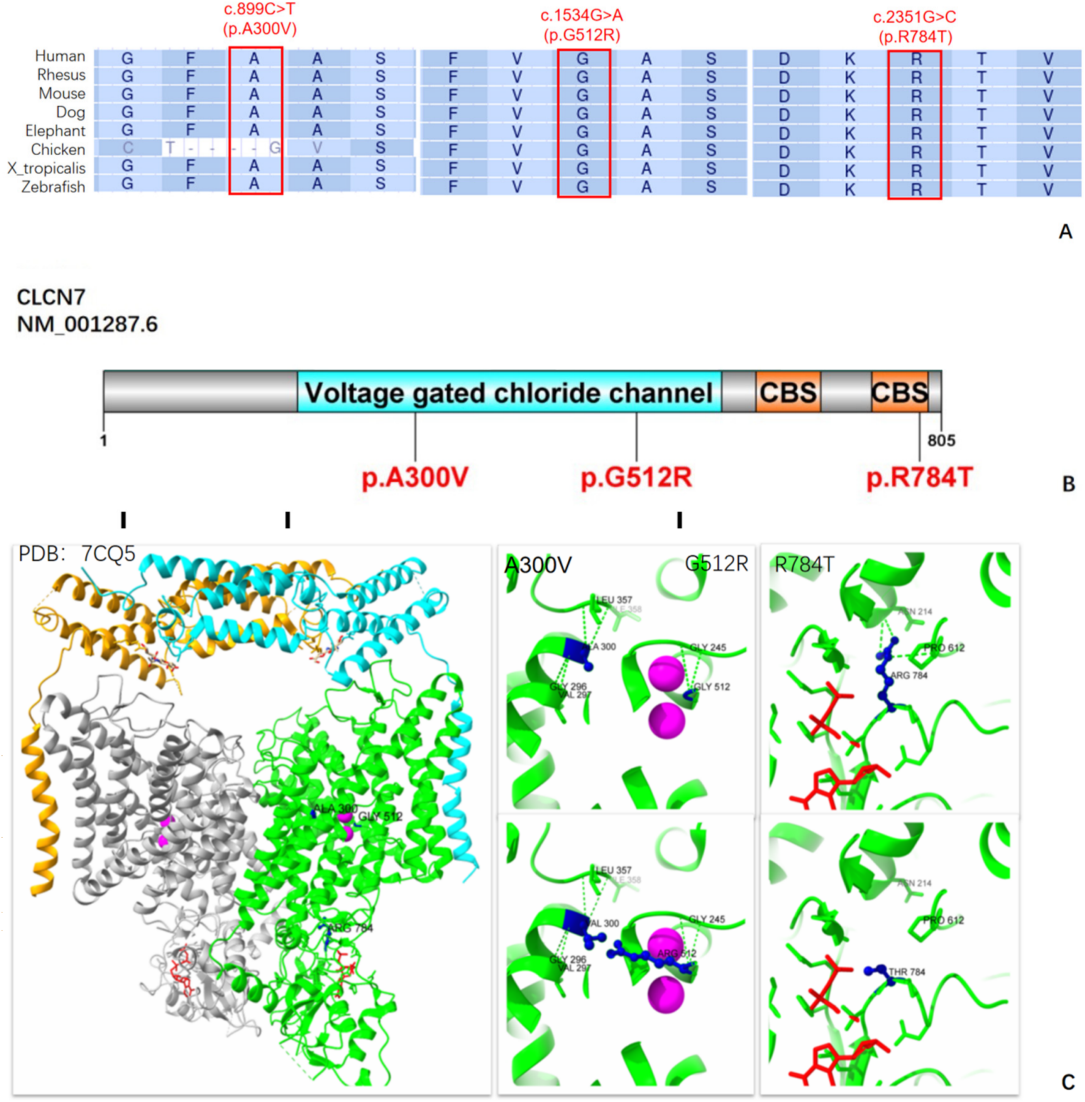
Molecular analysis of the variants A300V, G512R, and R784T on the CLC-7 protein. **(A)** A cross-species comparison of amino acid sequences revealed that the A300V, G512R and R784T variants are situated in a highly conserved region of the CLC-7 protein. **(B)** A schematic representation of the CLC-7 protein highlighting the locations of our *CLCN7* variants. CLC-7 protein include a transmembrane domain and a cytoplasmic C-terminal region that contains two CBS (cysteine β-synthase) domains. **(C)** 3D Structure of the human *CLCN7*-OSTM1 complex with ATP. The two ClC-7 subunits are represented in gray and green, and the two Ostm1 subunits are shown in orange and cyan. The position of the Cl^−^ ion is marked by a magenta sphere, ATP is shown in red, and our three variants are indicated in dark blue.

Multiple protein sequence alignment of Chloride Channel-7 (CLC-7) orthologs from eight species showed that c.2351G>C (p.R784T) was highly conserved, indicating the importance of this variant site. Although the c.899C>T (p.A300V) variant has been reported in a small cohort from Turkey (10) and c.1534G>A variant has been recorded in clinvar, protein modeling and functional validation of this variant have not been performed. Multiple protein sequence alignment suggested that c.1534G>A (p.G512R) is highly conserved, while c.899C>T (p.A300V) is only different in Chicken (Figure 3A). According to the 3D structure of the human CLC-7/OSTM1 complex, the p.R784T variant is situated in the cystathionine β- synthase 2 (CBS2) domain, where it disrupts three hydrogen bond interactions among Arg784, Asn214, and Pro612, potentially impairing the protein's structure and function (Figures 3B,C). Both p.A300V and p.G512R are located in the transmembrane domain of protein and near the Cl-conduction pathway. While the mutations Val300 and Arg512 do not alter the surrounding hydrogen bond interactions, their longer side chains resulting in greater steric hindrance may affect the stability of the spatial structure.

## 3 Discussion

The *CLCN7* gene, located on chromosome 16p13.3, consists of 25 exons encoding the 805-amino-acid CLC-7 protein. This protein forms a homodimer with two homologous monomers exhibiting reverse symmetry. Each monomer contains 18-intramembrane alpha helices, along with four Cl^−^ binding sites, and two CBS domains, crucial for endocytosis, lysosomal pathways, and ruffled borders of osteoclasts. It mediates chloride ion conduction and proton exchange within osteoclast lysosomes, maintaining the acidic environment necessary for bone resorption, and playing a vital role in osteoclast acidification (11). Variants in the *CLCN7* gene cause osteosclerosis, a condition marked by impaired osteoclast function (12). In this study, we identified a novel heterozygous missense variant c.2351G>C (p.R784T) in *CLCN7* gene of one Chinese family members in the proband and her asymptomatic mother. In addition, compound heterozygous variants c.899C>T (p.A300V) and c.1534G>A (p.G512R) in the *CLCN7* gene of another family were identified.

The clinical manifestations of osteopetrosis vary widely. The 2023 classification of osteopetrosis distinguishes subtypes based on genotype, separating them from high bone mass disorders caused by increased bone formation. ADO is the most common form, with ADOI characterized by skull thickening without fracture risk and ADOII affecting the spine and long bones with higher fracture susceptibility. Previously, *LRP5* variants were classified as ADOI but are now categorized under “osteosclerotic disorders” (13). The *CLCN7* gene is the main pathogenic gene for ADOII, first described by Albers-Schonberg in 1904 (14), with a penetrance of 56%–90% (15). The incidence of ARO is about 1 in 200,000 (16). *CLCN7* variants can also cause ARO, presenting early with severe symptoms like bone marrow failure, and IAO with milder symptoms such as minor trauma fractures and moderate anemia. Despite the rare involvement of the nervous system, visual impairment due to optic nerve injury can occur (17). *CLCN7*-related osteopetrosis also manifests with dental abnormalities root dysplasia, tooth eruption disorder, dental hypoplasia, malformed teeth, and enamel dysplasia (18). Disruption of CLC-7 expression can lead to severe lysosomal storage disorders, and neurodegeneration, including retinal atrophy (19).

Our study identified *CLCN7*-related ADO in patient 1 with a heterozygous variant inherited from her asymptomatic mother. Patient 1 was admitted after a head injury with recurrent nausea and vomiting and also had recurrent anemia, splenomegaly, fractures, left eye strabismus, and optic neuritis. However, a Y715C variant also located in the CBS domain reported by Nicoli et al. showed hypopigmentation, hepatosplenomegaly, and delayed myelination and psychomotor development in patients carrying this variant (20), which also indicated that the clinical characteristics of the variants may be significantly different even if they occur in the same functional domain. Although elevated tartrate resistant acid phosphatase (TRAP) levels are common in ADOII patients (21), their specificity requires further validation without correlating with disease severity. The final diagnosis depends on genetic testing, which is essential for evaluating treatment responses, prognosis, and risk of recurrence, and for distinguishing between different subtypes. We also identified *CLCN7*-related IAO in patient 2 and his sister, both with compound heterozygous variants. The older sister had skeletal abnormalities, optic nerve atrophy, and could only see nearby larger objects at age 17 years, while patient 2 had increased bone density discovered during a routine examination.

The *CLCN7* gene has at least 162 variants documented in the HGMD database, including 112 missense, 16 splice sites, 13 frameshift, and 9 nonsense variants. These variants are predominantly found in patients with ADOII and ARO. Hotspot variants like p.G215R, p.P249l, p.R286W, and p.R767W cluster near the intracellular gates of the CLC-7 channel dimer. In our study, the identified variants, c.2351G>C (p.R784T), c.899C>T (p.A300V), and c.1534G>A (p.G512R), are located in highly conserved regions of the CLC-7 protein. In family 1, the proband with ADOII carried the heterozygous missense variant c.2351G>C, resulting in the p.R784T substitution located in the CBS2 domain. This domain is evolutionarily conserved and is found in archaea, prokaryotes, and eukaryotic proteins. A variety of human diseases (homocystinuria, retinitis pigmentosa, hypertrophic cardiomyopathy, myotonia congenital, etc) are associated with amino acid mutations in the CBS domain (22). This variant disrupted crucial hydrogen bond interactions involving Arg784, Asn214, and Pro612, however, a similar variant, p.R784W, has been associated with late-onset bone symptoms in a Han Chinese family, where two patients (46-year-old male and 15-year-old male) experienced fractures, while two others (67-year-old female and 37-year-old male) complained of bone pain (8). However, patient 1 (3-year-old girl) in our study had an earlier onset possibly linked to head trauma or younger age. The correlation between genotype and phenotype remains to be fully elucidated, and additional factors such as epigenetic modification, phosphorylation, and ubiquitination may influence CLC-7 protein function. Patient 2, who had IAO, harbored compound heterozygous missense variants c.899C>T and c.1534G>A, which affected the *α* helix structure within the membrane. This structural change potentially hindered chloride ion diffusion due to elongated side chains, possibly explaining the milder symptoms observed. The c.899C>T variant has been previously reported in homozygotes in two siblings with *CLCN7*-related intermediate autosomal recessive osteopetrosis (IRO) in Turkey (10). The older sibling presented with visual disturbances, recurrent fractures, and osteoarthritis at age 13, while the younger experienced only back pain and osteosclerosis, which resembles the mild condition of the patients in family 2. At the 2022 Pediatric Endocrine Society (PES) Annual Meeting, a 4-month-old girl with a history of NICU stay, cytopenia, seizures, and congenital anomalies was found to have a homozygous p.G512R variant due to paternal uniparental disomy (UPD) of Chromosome 16, alongside a Chr 15q11.2 deletion. This case highlights the complexity of chromosomal changes associated with *CLCN7* variants. Moreover, the potential progression of unaffected carriers to affected patients over time warrants further investigation. Further research is needed to understand the underlying mechanisms of these variants comprehensively.

At present, there is no systematic treatment for osteopetrosis, and there is no specific drug, and symptomatic treatment is mainly used. However, patients with severe hematopoietic failure should be transplanted with hematopoietic stem cells as soon as possible (2). It has been reported that the 5-year disease-free survival rate of ARO patients after HSCT is about 42%–62% (8). A preimplantation genetic diagnosis can prevent the birth of ARO children. Previous research results of our team showed that one patient of ARO with *TCIRG1* gene mutation had no nervous system damage, and the effect was significant after Matched Sibling Donor—Hematopoietic Stem Cell Transplantation (MSD-HSCT) transplantation (23). However, studies have shown that CLCN7-related ARO cannot reverse the damage to the central nervous system after transplantation due to the combination of severe and persistent central neuropathy (24). Relevant studies have shown that patients with ADOII and healthy carriers have worse disease during follow-up, and the risk of fracture and arthritis is high. In our study, patient 2 had progressive bone marrow failure and extramedullary hematopoietic performance during the follow-up. After HSCT, extramedullary hematopoietic symptom was significantly improved. It was further proved that the clinical phenotype of patients with the *CLCN7* genotype had strong heterogeneity.

In summary, we identified a novel mutation c.2351G>C (p.R784T), coupled with protein function model prediction and homology analysis for two other variants already reported in the clinvar database [c.899C>T (p.A300V) and c.1534G>A (p.G512R)]in *CLCN7* gene, which expanding the mutation spectrum linked to this condition and enhancing our understanding of *CLCN7*–related osteopetrosis. Its clinical manifestations vary widely, predominantly classified as ADOII, but also including ARO and IAO. There is a correlation between clinical phenotype and genotype. The analysis results of variation data provided by us can be used as the theoretical basis for subsequent research, and the detection of more pathogenic variation sites will enable us to accurately diagnose, screen and classify osteopetrosis more accurately and quickly in the future.

## Data Availability

The datasets presented in this study can be found in online repositories. The names of the repository/repositories and accession number(s) can be found in the article/Supplementary Material.
