# Supplementary material for: Identification of a novel mutation in the CLCN7 gene in pediatric osteopetrosis: case report
Source: Front Pediatr. 2025 Apr 10;13:1549961. doi: 10.3389/fped.2025.1549961 (PMC12020480; doi:10.3389/fped.2025.1549961)
Supplement: Supplementary file 1 [file Datasheet1.pdf]

Methods

Patients and their families

Two children with osteopetrosis and their families, admitted to Anhui Provincial Children's Hospital between 2017 and 2020, were selected for this study (Figure S1). Both patients exhibited typical X-ray manifestations of osteopetrosis, including systemic osteosclerosis, "bone in bone", "sandwich vertebra", "halo sign", and "club-like thickening". Informed consent was obtained from the parents of all participating children, and the study was approved by the hospital's Ethics Committee. The follow-up period extended until March 31, 2024.

Laboratory and imaging examinations

The patient's medical history, birth history, feeding history, growth history, and family history were collected, and a related physical examination was performed. Laboratory examinations include routine blood tests, calcium (Ca), phosphorus (P), lactate dehydrogenase (LDH), creatine kinase (CK), creatine isoenzymes, serum alkaline phosphatase (ALP), liver and kidney function tests, bone marrow cytology, etc. Imaging examinations included anterior-lateral thoracolumbar radiographs, anterior-pelvic radiographs, X-rays of affected areas, head and chest CT scans, and head MRI scans, etc.

Whole exome sequencing

2mL of peripheral blood (EDTA anticoagulated) was extracted from pediatric patients and their family members. Genomic DNA was extracted using a Blood Genomic DNA Midi Kit (CWBIO), following the kit's instructions. The DNA samples were quality controlled using a Qubit 2.0 fluorometer and 0.8% agarose gel electrophoresis. For liquid-phase hybridization capture, the IDT xGen® Exome Research Panel v1.0 acquisition probe and gDNA library sequence were used. A whole exon library was constructed, covering the coding region and some non-coding regions of 19396 genes in the human genome, with a capture interval size of 51Mb. High-throughput sequencing (PE150) was performed using Illumina's NovaSeq 6000 series sequencers, achieving a target sequence coverage of more than 99%. The sequencing was conducted at Beijing Chigene Translational Medicine Research Center Co., Ltd.

Variant analysis

The raw sequencing data underwent initial quality control using Fastp software to trim adapters and remove low-quality reads. Sequence alignment to the Ensemble reference genome GRCh37/hg19 was performed using the Burrows-Wheeler Aligner (BWA) software. GATK software was utilized for single nucleotide variations (SNVs) and small insertions and deletions (Indels) analysis. Detected SNVs and Indels were further filtered based on sequencing depth and quality metrics to obtain high-quality and reliable variants. Annotation of minor allele frequencies (MAFs) and assessment of pathogenicity for each identified gene variant were performed using the online system developed by Chigene (www.chigene.org). To enhance specificity, common variants (allele frequency > 1%) found in public databases such as dbSNP, 1000 Genomes, ExAC, ESP, and gnomAD were excluded. The remaining variants were subjected to predictive analysis using tools including Provean, SIFT, Polyphen2-HVAR, Polyphen2-HDIV, M-Cap, Revel, and Mutationtaster. Assessments of potential impacts on splicing were conducted using SpliceAI and MaxEntScan. Clinical phenotypes of patients were delineated using terms from the Human

Phenotype Ontology (HPO, https://hpo.jax.org/) to assist in identifying disease-causing variants. We conducted a pathogenicity assessment of these variants based on their classification, population carrier frequency, consultation with OMIM, HGMD, and ClinVar databases, and adherence to the American College of Medical Genetics and Genomics (ACMG) guidelines for variant interpretation.

Sanger sequencing

PCR was used to amplify the target sequence of the *CLCN7* gene using primers detailed in Table 1. The PCR reaction conditions included an initial denaturation at 95°C for 5 min, followed by 30 cycles of denaturation at 95°C for 30 S, annealing at 60°C for 30 S, and extension at 72°C for 30 S, with a final extension step at 72°C for 10 min. Each PCR reaction was conducted in a 50 μL volume. Sequencing was performed using an ABI 3730XL sequencer with the original PCR primers. DNASTAR software was used for gene sequence analysis and comparison, referencing the *CLCN7* transcript NM_001287.6.

Bioinformatics Analysis

Multiple sequence alignments of the CLCN7 protein sequence (NP_001278.1) were conducted using the UCSC Genome Browser on Human (GRCh37/hg19). Protein domain prediction and structure analysis for CLCN7 were performed using the Uniprot database. A schematic diagram illustrating protein domain structures with functional motifs and sites was generated using DOG 2.0. Furthermore, the protein's 3D structure (PDB ID 7CQ5) was analyzed and visualized using ChimeraX (https://www. cgl. ucsf. edu/ chime ra/) to evaluate the impact of the identified missense variant.

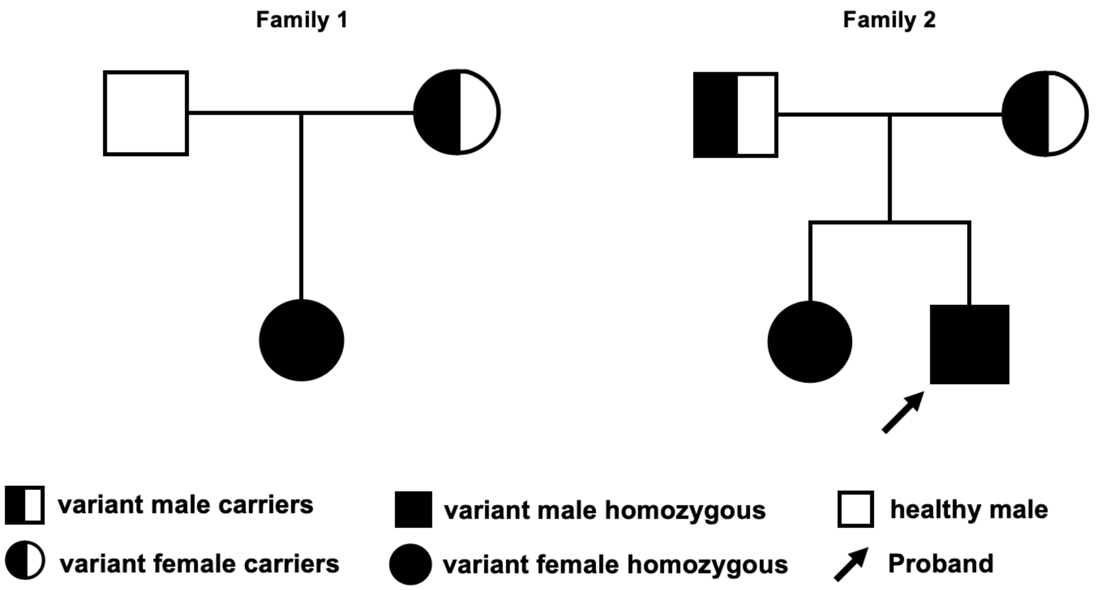

Figure S1. Pedigree of the two families in the present study. Squares and circles indicate males and females. Arrows represent the proband. Filled symbols show affected individuals.

Table S1. The PCR primers of *CLCN7* gene variants

Primers	Primer sequence	PCR product (bp)	Variants
CLCN7-1F	CAGGAATCAGAGCTGCTGACTCGGTT	1293	c.899C>T
CLCN7-1R	ACCAAATTCTCAACCTGGGCCTTAAGC		
CLCN7-2F	CCCGCTACCCTGTCGCCGATCCTT	1420	c.1534G>A<br>c.2351G>C
CNCN7-2R	TGCAGGGTGCTCGCCATTGCCACT		
